# Expansion/Facemask Treatment of an Adult Class III Malocclusion

**DOI:** 10.1155/2014/270257

**Published:** 2014-02-19

**Authors:** Gregory W. Jackson, Neal D. Kravitz

**Affiliations:** ^1^Department of Orthodontics (M/C 841), College of Dentistry, University of Illinois at Chicago, 801 S. Paulina Street, Chicago, IL 60612, USA; ^2^25055 Riding Plaza, No. 110, South Riding, VA 20152, USA

## Abstract

The orthodontic treatment of class III malocclusion with a maxillary deficiency is often treated with maxillary protraction with or without expansion. Skeletal and dental changes have been documented which have combined for the protraction of the maxilla and the correction of the class III malocclusion. Concerning the ideal time to treat a developing class III malocclusion, studies have reported that, although early treatment may be the most effective, face mask therapy can provide a viable option for older children as well. But what about young adults? Can the skeletal and dental changes seen in expansion/facemask therapy in children and adolescents be demonstrated in this age group as well, possibly eliminating the need for orthodontic dental camouflage treatment or orthognathic surgery? A case report is presented of an adult class III malocclusion with a Class III skeletal pattern and maxillary retrusion. Treatment was with nonextraction, comprehensive edgewise mechanics with slow maxillary expansion with a bonded expander and protraction facemask.

## 1. Introduction

The orthodontic treatment of Class III malocclusion with a maxillary deficiency is often treated with maxillary protraction either with or without maxillary expansion [1–4]. Studies on both humans and experimental animals have demonstrated the orthopedic advancement of the maxilla. These studies have shown that a significant component of skeletal class III malocclusion includes maxillary retrusion in combination with a normal or mildly prognathic mandible [5–17]. Skeletal and dental changes have been documented in these studies which have combined for the protraction of the maxilla and the correction of the class III malocclusion. Is there an ideal time to treat a developing class III malocclusion? Just a few studies have examined the effect of age on maxillary protraction therapy. Takada et al. [9] examined 61 Japanese female patients with class III malocclusion, divided into three groups (7 to 10 years, 10 to 12 years, and 12 to 15 years). They concluded that a greater orthopedic effect was observed when therapy was applied before or during the pubertal growth spurt (7 to 12 years). Baik [14] studied maxillary expansion and protraction in 47 Korean subjects, divided into three groups (<10 years, 10 to 12 years, and 12 years or older). He concluded that age did not show any statistically significant difference in treatment effects of expansion/facemask therapy. Braun [18] studied 63 subjects aged 4–13 and found that expansion/facemask therapy produces dentofacial changes that combine to improve class III malocclusion. They reported that, although early treatment may be the most effective, facemask therapy can provide a viable option for older children as well. But what about young adults? Can the skeletal and dental changes seen in expansion/facemask therapy in children and adolescents be demonstrated in this age group as well, possibly eliminating the need for orthodontic dental camouflage treatment or orthognathic surgery? 

## 2. Case Report

**Figure 1:**
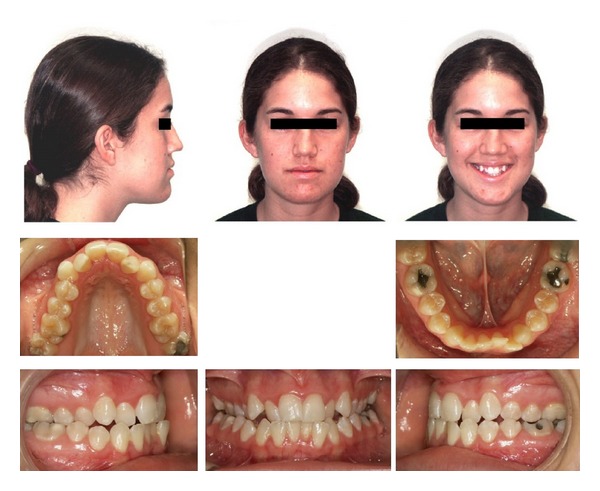
Initial composite.

**Figure 2:**
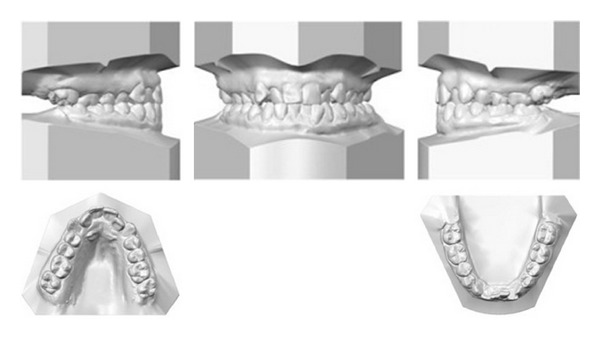
Initial models.

**Figure 3:**
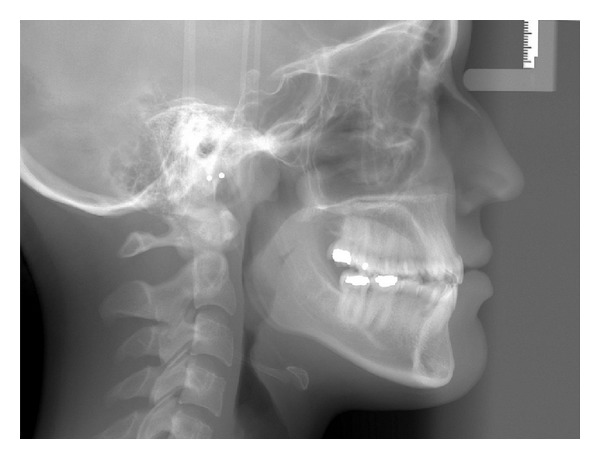
Initial lateral cephalometric radiograph.

**Figure 4:**
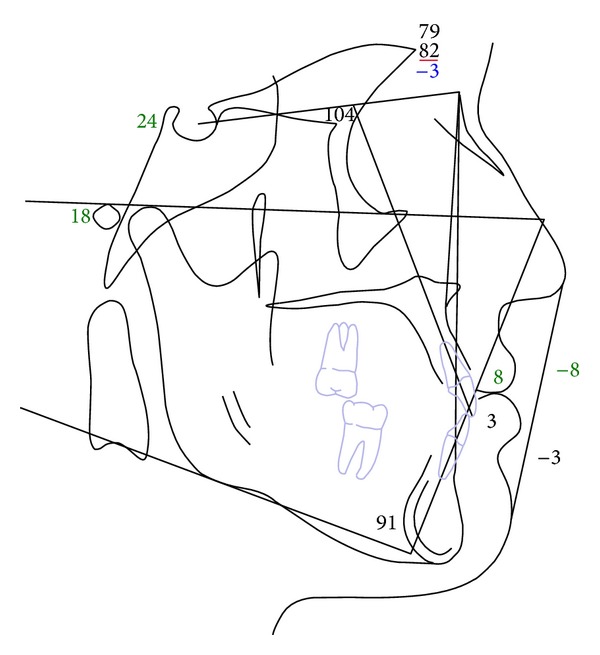
Initial lateral cephalometric tracing.

**Figure 5:**
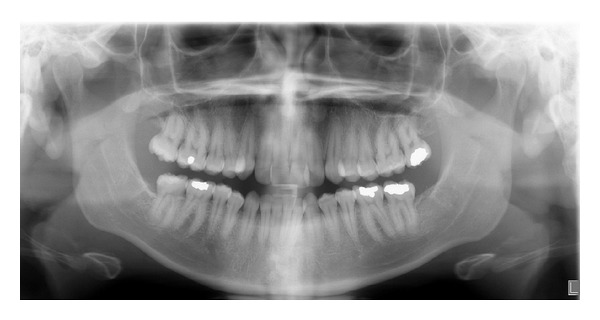
Initial panoramic radiograph.

**Figure 6:**
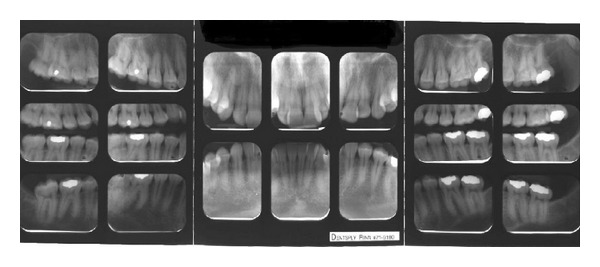
Initial full mouth radiographs.

**Figure 7:**
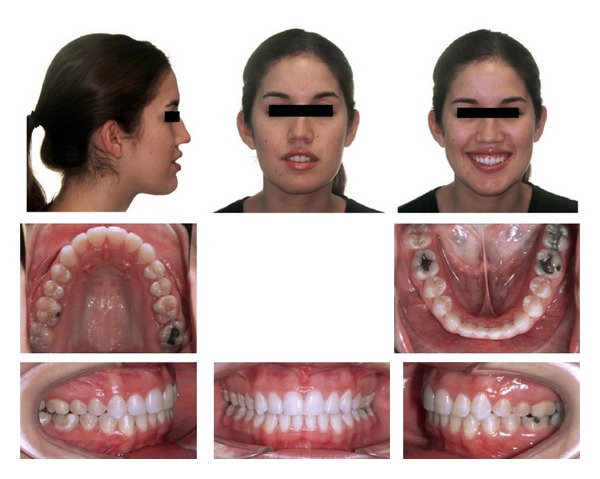
Final composite.

**Figure 8:**
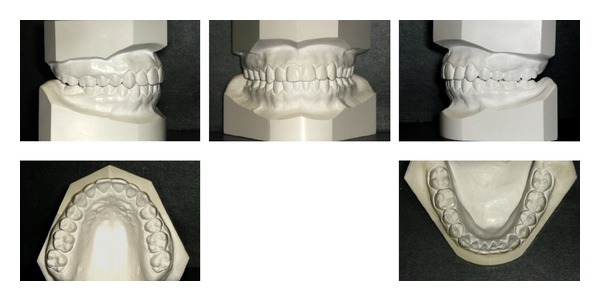
Final models.

**Figure 9:**
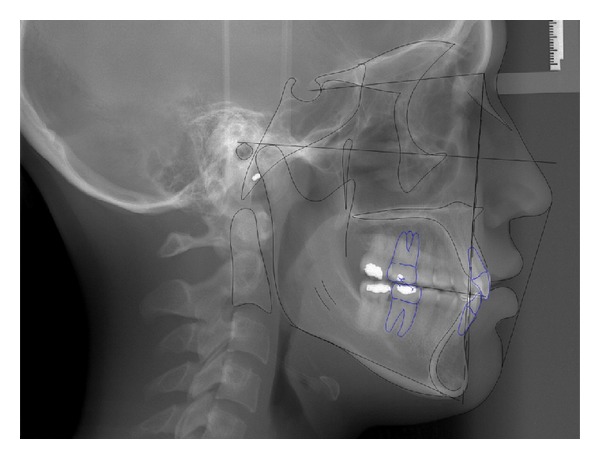
Final lateral cephalometric radiograph.

**Figure 10:**
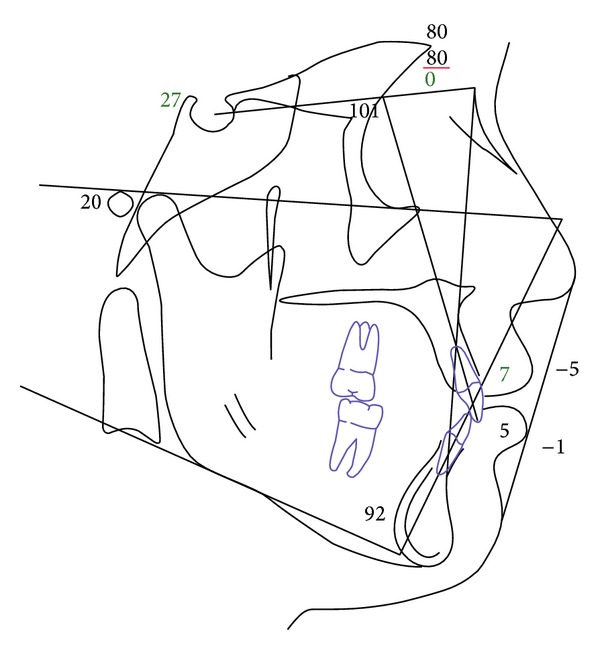
Final lateral cephalometric tracing.

**Figure 11:**
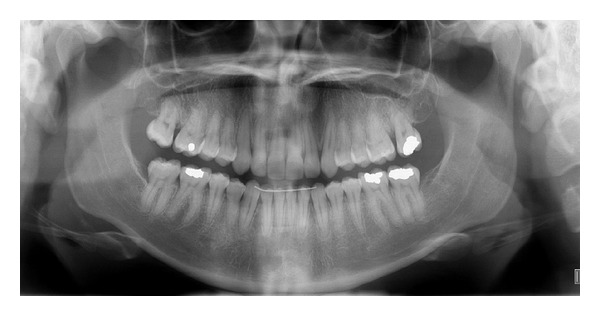
Final panoramic radiograph.

**Figure 12:**
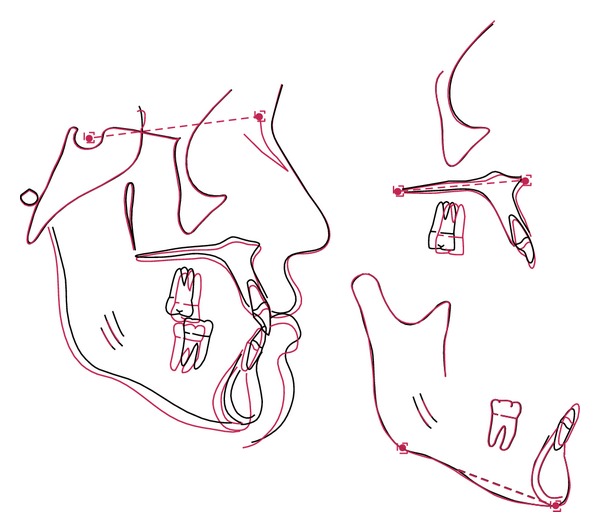
Superimposition.

A 19-year-1-month-old Caucasian female presented with a chief complaint of “I do not like my underbite.” Her medical history was noncontributory. She had a symmetrical, mesofacial face and a concave soft tissue profile (Figure 1). Her upper lip was slightly retruded. She presented with maxillary hypoplasia and flat malar eminences. She had a permanent dentition with class III malocclusion in both molars and canines (Figure 2). The maxillary arch was tapered with moderate crowding and the mandibular arch was ovoid with moderate crowding. All third molars had been previously removed (Figures 5 and 6). She had both anterior and bilateral posterior crossbites. Her maxillary midline was centered to her face and the mandibular midline was 1 mm to her right with an overjet of 0 mm and an anterior open bite tendency. The cephalometric analysis indicated a retrusive maxilla and protrusive mandible (Figures 3 and 4). No growth was anticipated in this patient. Both TMJs were normal and no habits were apparent. Dental treatment objectives included resolving upper and lower crowding, achieving both Class I molar and canine relationships, and correction of the anterior and posterior crossbite and midlines. Facial treatment objectives were to increase her upper lip fullness and malar eminences. Treatment was with nonextraction, comprehensive edgewise mechanics with slow maxillary expansion with a bonded expander and protraction facemask to develop the patient's cheek bones and create a fuller face which was not felt to be attainable with class III elastics alone. The acrylic of the maxillary expander covered the first premolars through the second molars bilaterally. The patient had an excellent attitude toward treatment and was highly compliant. During the slow expansion (1 turn or 1/4 mm per day) the midpalatal suture opened, and rapid expansion (two turns or 1/2 mm per day) was continued from that point on. The expander was activated with a total of 5 mm over a period of 16 days and then stabilized with light cured composite. The facemask had forehead and chin anchorage pads secured to a vertical bar. The 14 oz 1/2 inch elastics used to deliver a force of 500 gm bilaterally were attached to hooks on the expander in the maxillary canine areas and a crossbar on the facemask with a downward direction of about 30 degrees. The patient agreed to four months of protraction (during the summer while out of school) for 18–20 hours per day. At the completion of expansion and protraction, the patient's molar and canine relationships were overcorrected to a slight class II tendency. Overall treatment and malar development were highly successful (Figures 7, 8, and 11). The patient's malocclusion was primarily corrected with maxillary protraction and clockwise/vertical rotation of the mandible (Figures 9, 10, and 12). A point moved forward 1.5 mm and downward 2 mm (Figure 12). Pogonion moved 2 mm down and 2 mm backward. Her upper lip moved forward 2 mm. The patient was incredibly pleased with the overall results. She exhibited great cooperation with her treatment, especially with the wearing of the facemask and intraoral elastics. This undoubtedly greatly contributed to her successful treatment result. An honest evaluation of the expected patient cooperation is always important in orthodontic treatment but especially with a teenager with several different patient compliance dependent appliances. She was retained with a maxillary Hawley and fixed mandibular canine to canine retainer. Treatment was completed in 17 months and proved to be stable following the active treatment.

## 3. Discussion

The only drawback of this approach (protraction therapy versus surgical) is that it increased the vertical dimension and the gingival exposure increased by 3 mm. Perhaps the patient would have benefited from maxillary protraction more closely through the center of resistance of the maxilla as described by Braun and Marcotte [18–20]. Another treatment option could have been bone-anchored maxillary protraction (BAMP) as described by de Clerck et al. [21]. They reported successful maxillary protraction in the late mixed or permanent dentition age of 10–14 years. This approach requires surgery for placement and removal of the miniplates and increased costs for the miniplates and surgery.

This patient facemask/expansion therapy affected many areas of her dentofacial complex. Skeletal change was primarily a result of anterior and vertical movement of the maxilla. Significant changes in mandibular position also contributed to the class III correction. The downward and backward movement of the chin expressed in this patient have been described by Ishii et al. [7] and Takada et al. [9], with maxillary protraction and chin cup, and Ngan et al. [13] and Nartallo-Turley and Turley [2] and using palatal expansion with a facemask. Various soft tissue changes combined to improve the patient's class III profile. Her profile is becoming more convex due to forward movement of the upper lip and retraction of the lower lip, soft tissue pogonion moving back and menton moving down as described by Kapust et al. [17].

## 4. Conclusion

This case demonstrates that, given excellent patient cooperation, it is possible to treat an adult class III malocclusion with maxillary expansion and a protraction facemask.
